# Central Giant cell tumor of jaw bone in child: A case report

**DOI:** 10.1016/j.ijscr.2021.01.007

**Published:** 2021-01-06

**Authors:** Ouassime Kerdoud, Rachid Aloua, Faiçal Slimani

**Affiliations:** a Faculty of Medicine and Pharmacy, Hassan II University of Casablanca, B.P 5696, Casablanca, Morocco; b Oral and Maxillofacial Surgery Department, CHU Ibn Rochd, B.P 2698, Casablanca, Morocco

**Keywords:** Central giant cell tumor, Clinical features, Recurrence rate, Surgical approach, Mandibular disease

## Abstract

**Introduction:**

Central giant cell tumors (CGCTs) bones are uncommon benign bone tumors in the jaw.

**Presentation of case:**

The authors report a pediatric patient who was diagnosed with an aggressive central giant cell cyst of the mandible and its management. The CGCT usually occurs in adolescents patients from 5 to 10 years of age. An 8-year-old female patient presented with progressive non-pulsatile swelling of the mandible. Intraoral examination revealed mobility of teeth, associated with gingival swelling.

**Discussion:**

Common sites of giant cell tumors are the epiphysis of the long bones. Giant cell tumors accounted for 4–7% of all primary bone tumors. The mandible localization is exceptional, the main treatment for giant cell tumors is surgical excision and different surgical approaches had been reported in the literature thus depending on the size of the cyst, localization, and experience of the surgeon.

**Conclusion:**

Giant cyst tumor of the jaw is rare and less common in childhood. The clinical behavior is very diverse. The main treatment is surgical removal and follow-up to reveal recurrence.

## Highlights

• Giant cyst tumor of the jaw is rare and less common in childhood.
• The clinical behavior is very diverse.
• The main treatment for giant cell tumors is surgical excision.
• Follow-up is necessary to reveal recurrence.
• A major complication of surgical removal of the large cyst is iatrogenic fracture of mandible.

## 1 Introduction

Central giant cell tumors (CGCTs) bones are uncommon benign bone tumors in the jaw [1,2], 4–7% of all primary bone tumors [3]. CCTG is characterized by local aggressiveness and can be transformed into malignant CGCT with the risk of pulmonary metastasis. The etiology of giant cell tumors remains uncertain. The incidence shows a predilection for the female sex. The curative treatment for these tumors is surgical curettage or resection, undesirable damage of the teeth or tooth germs is unavoidable [4] and surgical removal may lead to rupture of the cyst or iatrogenic fractures. The local recurrence rates were as high as 25%.

## 2 Case report

Our work is a single case report and has been reported in line with the SCARE criteria [5].

A healthy 8-year-old girl presented with painless slow-growing swelling of the jaw that had persisted for 3 years, associated with dental loss. She was referred to our department's consultation for specialized care. No other personal or family history was raised during the patient interrogation. Clinical examination revealed large tender swelling without sensory disturbances and incompetent lips with a protruding lower lip. In the vertical direction, the lower third part of the face is larger than the other parts.

**Fig. 1:**
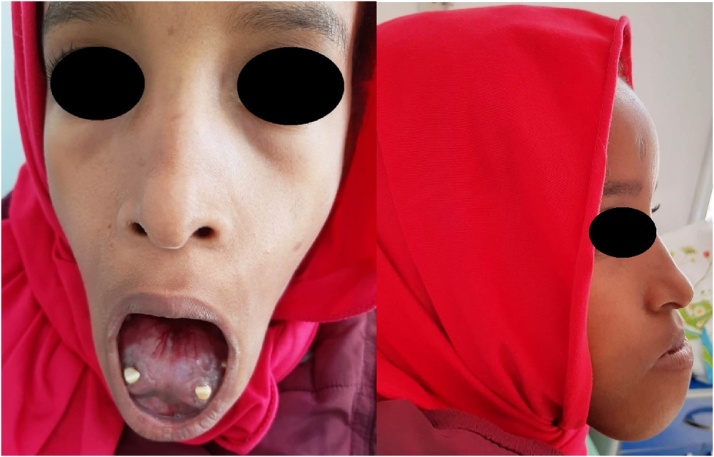
Preoperative clinical picture of the patient showing swelling of the jaw.

On intra-oral examination, she had hard swelling of about 50 mm, non-pulsatile, displacement of teeth leading to malocclusion (Fig. 1).

**Fig. 2:**
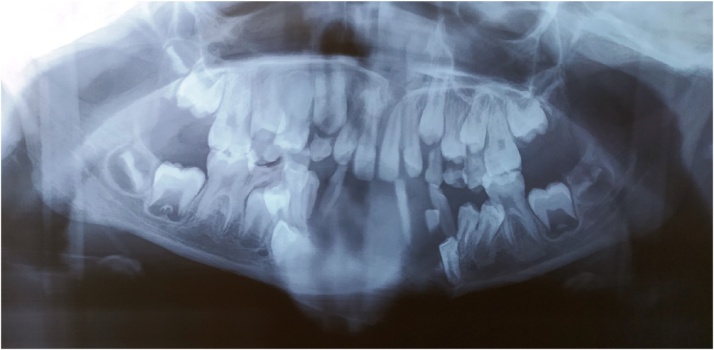
The panoramic dental x-rays showing central opacity with tooth displacement.

The panoramic dental x-ray showed a lesion of the mandible not well defined, tooth displacement, and root resorption (Fig. 2)

**Fig. 3:**
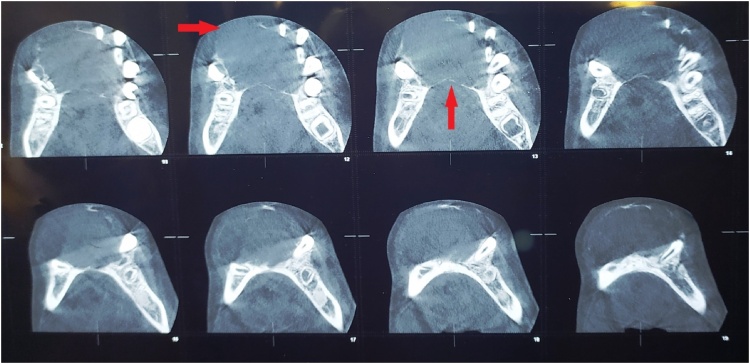
CT scan (axial cut) picture of the patient showing iso-dense cystic mass (arrow).

**Fig. 4:**
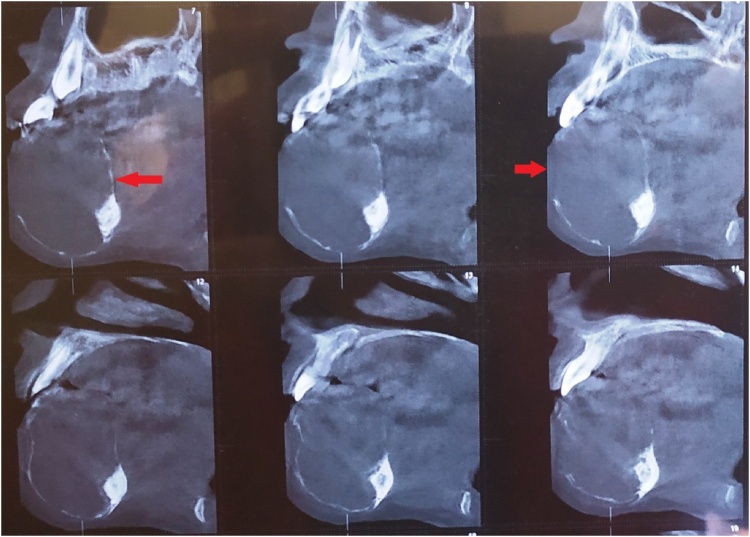
CT scan (sagittal cut) showing the mandibular mass (arrow).

The computed tomography (CT) was performed and revealed an iso-dense large destructive cystic mass 34 × 43 mm in an eccentric position of the mandible, pushing the tongue back, absence of peripheral sclerosis, trabeculation, and cortical perforation (Figs. 3, 4).

Endocrinology assessment was done to check hyperparathyroidism: calcium, parathyroid hormone, and phosphorus were at a normal level.

A biopsy concluded at a central giant cell tumor (CGCT).

Surgical intervention was performed by the chief professor of our department.

The patient has received under general anesthesia; the intra-oral approach of the cyst was done.

Intra-operatively, a unilocular cystic mass without liquid content was excised.

There was, however, a rupture of the external wall of the mandible cortical without iatrogenic fracture.

The patient received antibiotics and antalgics daily for 8 days.

The pathology of the surgical material concluded at the central giant cell tumor.

**Fig. 5:**
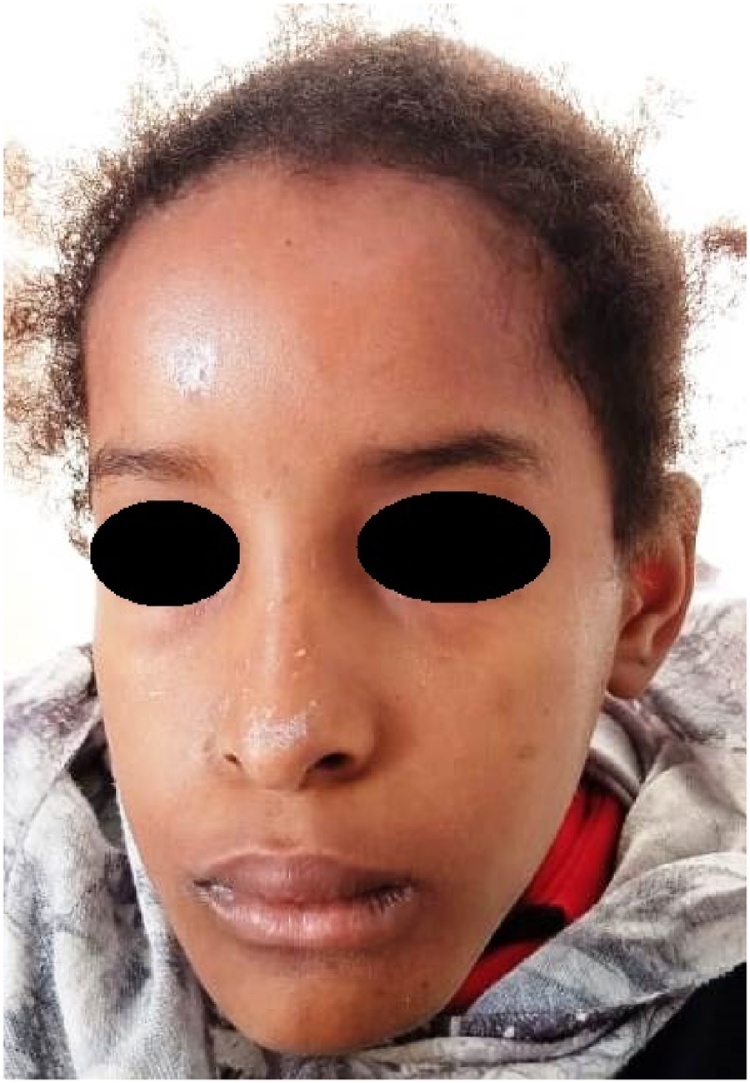
Postoperative picture.

Postoperative periods were favorable with the disappearance of swelling and advised to oral rehabilitation (Fig. 5).

The patient was followed up in our specialized consultation; any clinical signs that appeared were mentioned on the patient's discharge form.

Routine follow-up 3, 6, and 12 months later showed no signs of recurrence.

## 3 Discussion

Common sites of giant cell tumors are the epiphysis of the long bones. Giant cell tumors accounted for 4–7% of all primary bone tumors. The mandible localization is exceptional [3].

The most frequent clinical findings are swelling, local pain, tender mass, and pathological fractures [4].

Aggressive lesions are defined by the presence of one or more of the following signs: pain, paresthesia, root resorption, rapid growth, cortical perforation, and a high recurrence rate after surgical curettage [6,7].

Differential diagnoses are aneurysmal bone cyst, chondroblastoma, giant Cell-rich osteosarcoma, and brown tumor of hyperparathyroidism [8,9].

In the present case patient presented with progressive swelling of the jaw with dental loss, hyperparathyroidism was excluded in this case. However, due to the exposure site, most cases of giant cell tumors disease are usually diagnosed early in childhood but in this case, the late diagnosis is caused by the lack of accessibility of the patient.

The main treatment for giant cell tumors is surgical excision and different surgical approaches had been reported in the literature thus depending on the size of the cyst, localization, and experience of the surgeon [10].

A major complication of surgical removal of the large cyst is the iatrogenic fracture of the mandible [11].

The aggressive central giant cell tumors are less common than the literature has suggested. A strong association between multiple lesions and neurofibromatosis type 1 and Noonan syndrome has been shown to strongly associate [12].

Given the wide range of clinical behaviors, some lesions may simply be reactive in origin while in other cases; a yet undiscovered genetic abnormality may play a contributing role.

## 4 Conclusion

Giant cyst tumor of the jaw is rare and less common in childhood. The main treatment is surgical removal and follow-up to reveal recurrence. Surgeons must be aware of the variety of clinical behavior of these tumors with the risk of iatrogenic fractures in large tumor surgical resection. Although they are histologically similar, it is questionable whether all GCCGs have the same etiology.

## Declaration of Competing Interest

The authors report no declarations of interest.

## Funding

The authors declared that this study has received no financial support.

## Ethical approval

Written informed consent was obtained from the patient for publication of this case report and accompanying images. A copy of the written consent is available for review by the Editor-in-Chief of this journal on request.

## Consent

Written informed consent was obtained from the patient for publication of this case report and accompanying images. A copy of the written consent is available for review by the Editor-in-Chief of this journal on request.

## Author contribution

Ouassime Kerdoud: Corresponding author writing the paper.

Rachid Aloua: writing the paper.

Faiçal Slimani: Correction of the paper.

## Registration of research studies

researchregistry6312.

## Guarantor

Ouassime Kerdoud.

## Provenance and peer review

Not commissioned, externally peer-reviewed.
